# Strong 3D *and* 1D magnetism in hexagonal Fe-chalcogenides FeS and FeSe *vs*. weak magnetism in hexagonal FeTe

**DOI:** 10.1038/s41598-017-03502-5

**Published:** 2017-06-13

**Authors:** David S. Parker

**Affiliations:** Oak Ridge National Laboratory, 1 Bethel Valley Rd., Oak Ridge, TN 37831 USA

## Abstract

We present a comparative theoretical study of the hexagonal forms of the Fe-chalcogenides FeS, FeSe and FeTe with their better known tetragonal forms. While the tetragonal forms exhibit only an incipient antiferromagnetism and experimentally show superconductivity when doped, the hexagonal forms of FeS and FeSe display a robust magnetism. We show that this strong magnetism arises from a van Hove singularity associated with the direct Fe-Fe *c*-axis chains in the generally more three-dimensional NiAs structure. We also find that hexagonal FeTe is much less magnetic than the other two hexagonal materials, so that unconventional magnetically-mediated superconductivity is possible, although a large T_*c*_ value is unlikely.

## Introduction

Since the seminal discovery of superconductivity with a *T*_*c*_ of 26 K^1^ in LaFeAsO_1−*x*_F_*x*_ nearly nine years ago, the scientific community has shown great interest in the iron-based superconductors, with many thousands of papers published on this topic. Excellent reviews may be found in refs 2–10. Many, though not all of these materials show antiferromagnetism in an orthorhombic or monoclinic phase which appears from a tetragonal phase as the temperature is lowered. Superconductivity, usually accompanied by the disappearance of the lower-symmetry phase, occurs upon doping. Many theoretical studies have therefore been done on these tetragonal phases, and substantial work has also been done on the tetragonal Fe chalcogenides FeS, FeSe and FeTe. In particular, monolayers of FeSe deposited on SrTiO_3_ substrates are reported^11^ to have *T*_*c*_ values exceeding 100 K. In addition, tetragonal FeS has recently been synthesized and also exhibits superconductivity^12^, with the details strongly dependent on preparation conditions.

However, FeS, FeSe and FeTe also exhibit a hexagonal NiAs form (there are additional structures for FeS which we do not explore here). In general this form has been far less studied. For FeS this form is known as *pyrotthite* ^13^ and it is in fact much easier to synthesize than the tetragonal version, while for the other two Fe-chalcogenides few studies have been done. Hence we have conducted a theoretical study comparing the two forms of these compounds.

**Figure 1:**
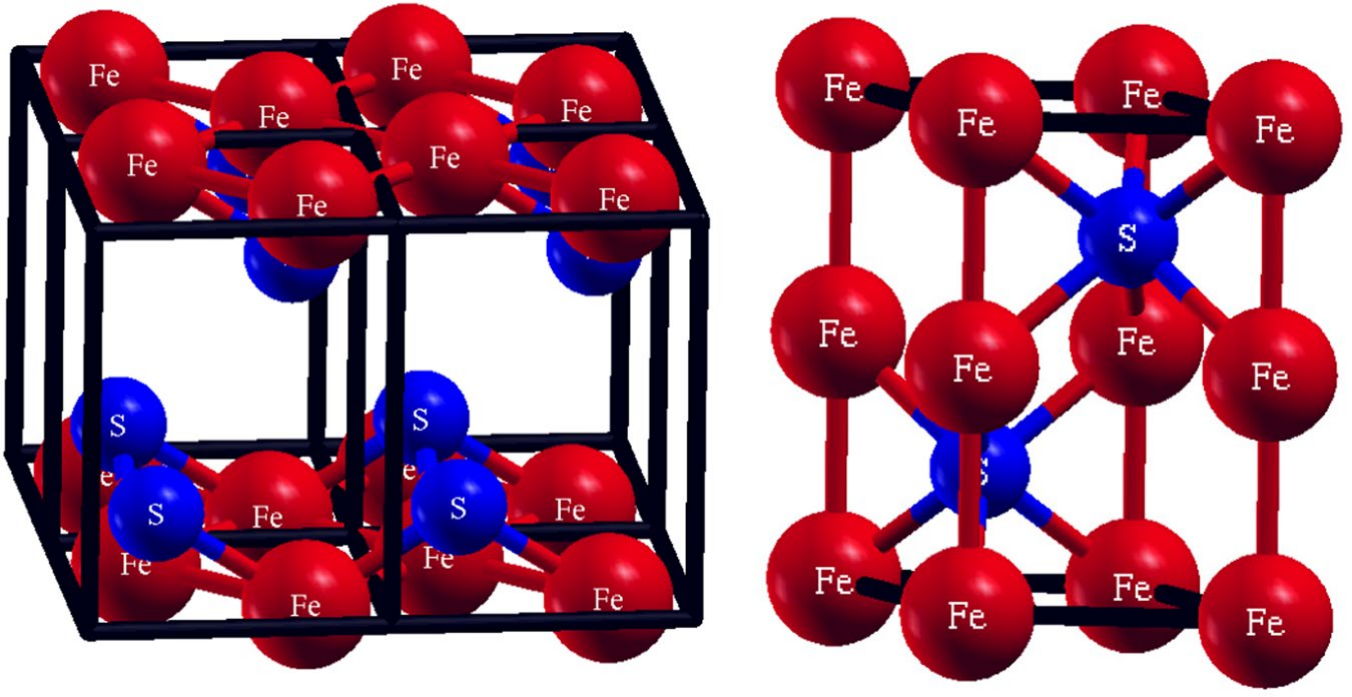
The two structures studied in this work. Left: the tetragonal structure; right, the hexagonal NiAs-structure. Pictures depict actual FeS structures.

We depict the essential structural difference between these two forms in Fig. 1. As is well known, the tetragonal structure (top) contains Fe layers in square coordination with an S or Se or Te) located at the apex of a pyramid. These layers are in general well-separated, which necessarily limits *c*-axis transport and is responsible for the basic two-dimensionality of these materials. For the hexagonal structure, the chalcogen (S in the figure) sits at an octahedral site with c-axis symmetry. There is therefore no “gap” as in the tetragonal structure and we anticipate a much more three dimensional structure. In addition, there are direct Fe-Fe *c*-axis chains and we therefore anticipate substantial c-axis transport. In fact for certain compounds this transport is predominant, yielding a *one*-dimensional character despite the generally three dimensional structure.

## Results

**Figure 2:**
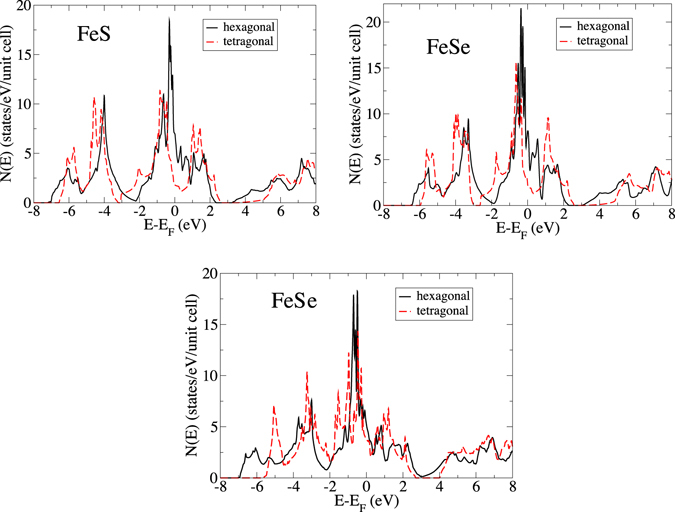
The calculated densities-of-states for FeS (top left), FeSe (top right) and FeTe (bottom), for the hexagonal (black solid line) and tetragonal (red dashed line) structures.

While the three hexagonal Fe-chalcogenides differ in significant ways, it turns out that the properties of FeS and FeSe are relatively similar, while those of FeTe differ significantly by being much less prone to magnetism. Let us begin the discussion with hexagonal FeS and FeSe, by way of comparison with the tetragonal forms. For these two materials, the essential electronic structure difference between the hexagonal and tetragonal forms can be seen in Fig. 2, which presents calculated densities-of-states (DOS) for the hexagonal and tetragonal polymorphs. The tetragonal chalcogenide *z* coordinate was relaxed for these calculations.

Figure 2 shows Fermi level DOS values *N*(*E*_*F*_) much greater -nearly 4 times higher - for the hexagonal than tetragonal forms. Specifically, *N*(*E*_*F*_)_*hex*_ for FeS and FeSe are 6.97 and 8.22/eV-unit cell, respectively. Nearly all of the DOS is attributed to the Fe atoms (not shown) Note also that there are two formula units per cell. Therefore, unlike the tetragonal forms, which are only marginally magnetic, the hexagonal forms are strongly magnetic. Assuming an exchange correlation *I* ^14^ for Fe of 0.5 eV, one finds Stoner parameters *IN*(*E*_*F*_) of 1.74 and 2.05, respectively, indicating a strong magnetic instability for both FeS and FeSe.

Indeed in the calculations we are able to stabilize numerous magnetic states, including a nearest-neighbor *c*-axis antiferromagnetic state (AF1), a ferromagnetic state, and other magnetic states, with energies several hundred meV per Fe below the non-magnetic state. Such large magnetic energies clearly indicate the formation of large magnetic moments whose magnitudes do not change substantially from one ordering pattern to another. Hence these are local moments, and the difference in energies between AF1 and the ferromagnetic state gives, in the mean-field approximation (in which the Neel temperature *T*_*Neel*_ is taken as 1/3 of this energy difference per Fe) a Neel temperature above room temperature.

Experimentally hexagonal FeS indeed exhibits magnetic order above room temperature, which effectively precludes the appearance of superconductivity in this system, to be compared to the tetragonal form, which exhibits a highly sample-dependent superconductivity around 4–5 K^12^. However, experimentally^15^ the magnetic order in hexagonal FeS is observed to be *ferri*magnetic as opposed to *antiferro*magnetic. The reason for this is that Fe vacancies typically form in this material, preferentially in every other layer, with the other atoms generally retaining their original moment. Hence a bulk sample develops a small average moment.

It is of interest to understand the reason for the much larger Fermi-level DOS, relative to the tetragonal polymorphs, in the hexagonal FeS and FeSe compounds. This is indeed surprising since the unit cell volume is much smaller for the hexagonal phase - the volume difference is of order 15 percent. Typically, smaller volumes (note that most of the DOS for both compounds near *E*_*F*_ is Fe) are associated with *more* atomic wave function overlap, therefore *more* bandwidth, *smaller* DOS and therefore *less* magnetism. *How then is it possible for the FeS and FeSe hexagonal polymorphs to be* **more** *magnetic than the tetragonal polymorphs*?

## Discussion

To answer the above question we first note from the DOS a van Hove singularity located just below the Fermi level, with an attendant high density-of-states at this point. While it is known that lower dimensionalities are more prone to van Hove singularities, with a one-dimensional DOS having a functional form at the band edge *E*_0_ of (*E* − *E*_0_)^−1/2^ and hence a large divergence in the DOS at the band edge, at first glance such a three-dimensional structure ought not support such a one-dimensional behavior.

A closer look at Fig. 1, however, reveals in the hexagonal structure the *c*-axis Fe-Fe chains that permit direct Fe-Fe transport. Since there is relatively little S character near E_*F*_, it follows that these chains are important in the electronic structure of this compound. Furthermore, while the hexagonal volume is significantly smaller, in FeS the hexagonal nearest-neighbor Fe-Fe distance, at 2.86 Å, is much larger than the 2.60 Å in the tetragonal structure, making for narrower bands. A similar situation applies for FeSe, with the hexagonal Fe-Fe distance of 2.95 Å, much longer than the tetragonal Fe-Fe distance of 2.67 Å, and for FeTe the respective distances are 2.83 and 2.70 Å. In addition, while in the tetragonal structure there is fourfold planar Fe-Fe coordination, and hence *two*-dimensional character, in this hexagonal structure there is twofold *axial* coordination, hence *one*-dimensional electronic structure.

It is of interest that the Fe-Fe nearest neighbor distance increase from the tetragonal to hexagonal structure is much smaller in FeTe, at 0.13 Å, than the 0.26 Å and 0.28 Å distance increase in FeS and FeSe. This smaller distance increase results from the larger atomic size of Te, relative to Se and S, which enlarges the planar tetragonal lattice constant (in the pyramidal site). In fact, despite this larger size, the hexagonal Fe-Fe nearest neighbor distances are *smallest* in FeTe. It is this fact, and the resulting increased wavefunction overlap, that is responsible for the decreased magnetic tendencies of hexagonal FeTe relative to FeS and FeSe.

**Figure 3:**
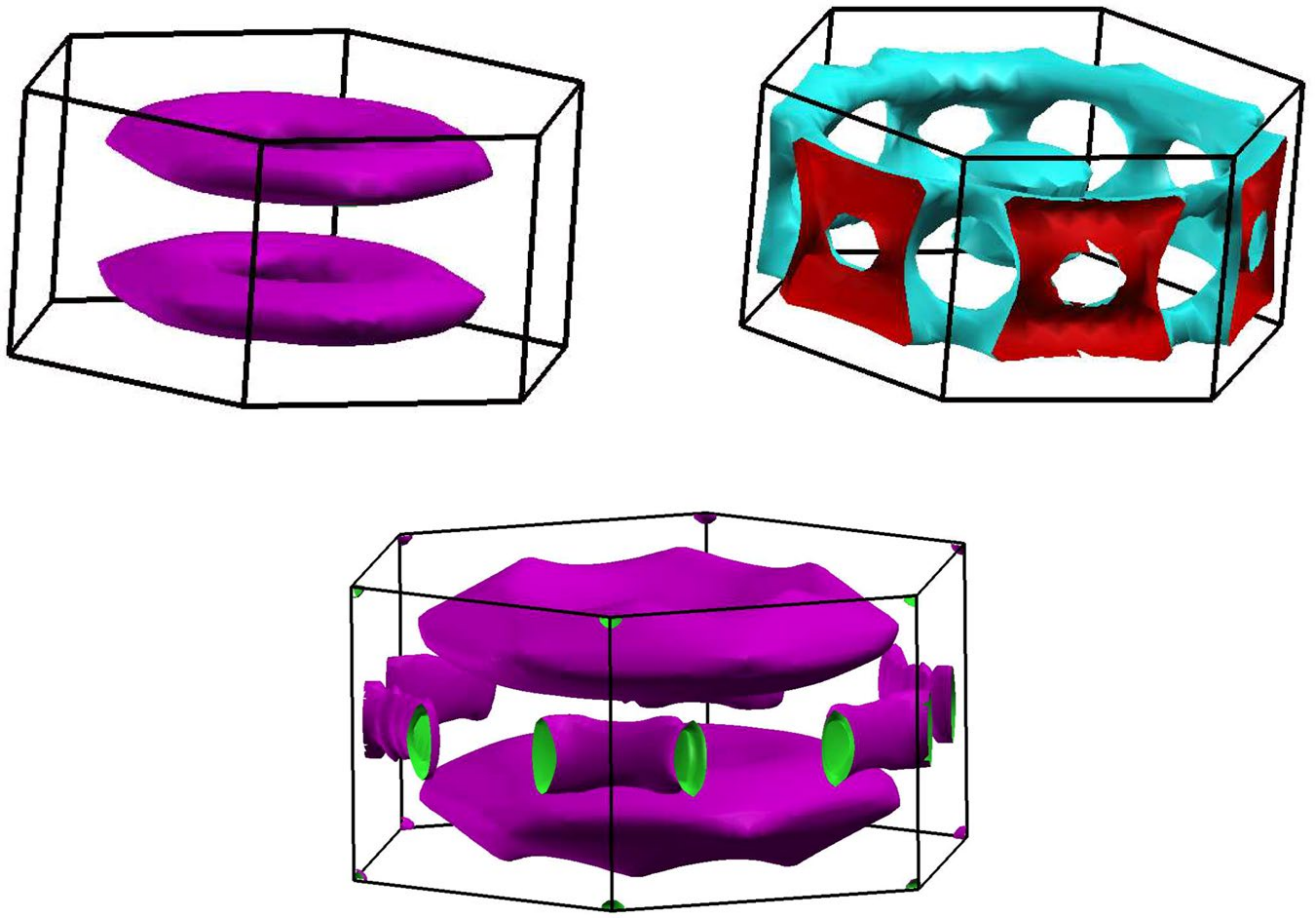
The calculated Fermi surfaces for hexagonal non-magnetic FeS - band 20, top left; band 21, top right. The bottom plot shows the FeS band 20 isoenergy surface for a Fermi level taken approximately 70 meV below the actual Fermi level. Brillouin zones are rotated slightly to better depict the Fermi surfaces. Note the predominant 1-dimensional character of band 20, the more three dimensional character of band 21, and the much larger band 20 isoenergy surface in the bottom plot, indicative of the nearness to the van Hove singularity.

We now examine in more detail the Fermi surface plots of the hexagonal (Fig. 3a–c) FeS polymorph. In Fig. 3a the Fermi surface of band 20 is plotted, showing a cylindrical plate-like feature parallel to the top (z) face of the Brillouin zone, exhibiting the one dimensional character. Interestingly, band 21 (Fig. 3b) has a very different character, consisting of rings connected by bridges, the structure mostly parallel to the outside faces of the Brillouin zone. Given that the motion of electrons described by the band 21 FS is mostly planar, we would expect Sulfur involvement in this electronic structure, from the physical structure. Indeed the proportion of the DOS that is Sulfur character (not shown) increases significantly as one increases energy across *E*_*F*_ and towards the more three dimensional band 21. Note that there is a band overlap of some 0.35 eV so that this transition occurs slightly below E_*F*_.

To show the nearness of FeS band 20 to the van Hove singularity, in Fig. 3c we plot the isoenergy surface of this band for the Fermi level lowered by some 70 meV from the actual value. We see a substantially enlarged “pancake”-like, nearly one-dimensional feature paralleling the basal plane but displaced approximately 3/4 *π*/*c* upwards from this plane towards the zone face. The substantial size increase is indicative of the nearness to the van Hove singularity. Tube-like features also appear in the basal plane but these carry less spectral weight.

**Figure 4:**
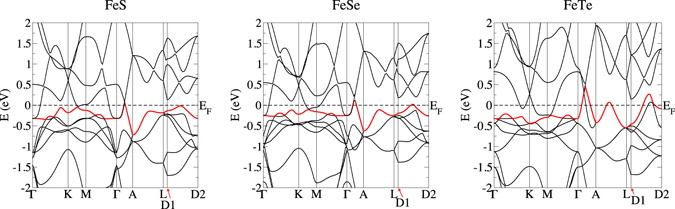
The calculated band structure plots for hexagonal FeS (left), FeSe (center) and FeTe (right). FeS band 20, and the corresponding FeSe and FeTe band 30, are shown in red. Except for D1 and D2, the notation is standard; D1 and D2 refer, respectively, to points 1/4 *π*/*c* below the L point (towards the basal plane), and a point this same distance below the A point at (0, 0, *π*/*c*). Hence the line D1-D2 parallels the basal plane, and for FeS and FeSe the van Hove singularity can be seen touching the Fermi level approximately halfway between these points, and from the generally very small dispersions along this direction. For FeTe the dispersion in this area is much larger and the van Hove singularity does not cut the Fermi level, partly accounting for the decreased magnetic character of hexagonal FeTe.

To better depict this feature, and its relative absence in FeTe, in Fig. 4 we depict, respectively the calculated band structure for FeS, FeSe and FeTe. The line from D1 to D2 parallels the basal plane and runs from a point 1/4 *π*/*c* below the L point to a point this distance below the A point. For FeS band 20 and FeSe band 30 (this band is shown in red) along this line there is a relatively small dispersion - 0.25 eV - indicative of the nearness to the singularity, and the bands just brush the Fermi level, significantly increasing the Fermi level DOS. For FeTe this dispersion is much larger, at 0.5 eV, and cuts the Fermi level sharply, thus making a much smaller contribution to the Fermi level DOS.

It is interesting to note that in all 3 materials there is a *secondary* van Hove singularity approximately 0.25 eV below the Fermi level (the flat bands in the Γ-K-M plane on the left hand side), corresponding to a nearly one-dimensional feature in the basal plane. It is this feature that contributes most strongly to the DOS peaks in Fig. 2. However, in this regime of momentum space this feature does not cut E_*F*_ and therefore does not contribute to the magnetic behavior. The beginnings of this feature (showing as basal plane tubes, which would broaden with decreasing Fermi energy) can be seen in Fig. 4c.

Returning briefly to the DOS plots of Fig. 2, we note in passing that the tetragonal versions of FeS, FeSe and FeTe also exhibit a DOS which rapidly varies in energy within an eV of the Fermi level, but the DOS peaks (which could conceivably also be considered van Hove singularities) are more nearly an eV away from E_*F*_, instead of the approximate 0.2 eV peak distance below E_*F*_ that hexagonal FeS and FeSe. These tetragonal DOS peaks therefore have less impact on the magnetic behavior of these polymorphs.

The notions of the dimensionality of FeS bands 20 and 21 can be more precisely quantified by considering the individual DOS of these two bands.

**Figure 5:**
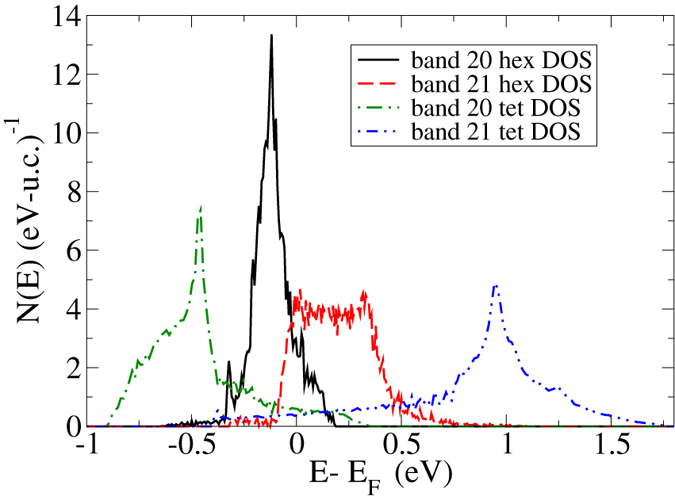
The calculated densities-of-states for the individual bands of non-magnetic FeS, as indicated. Note that for the tetragonal phase there are two more bands crossing E_*F*_, but we omit these in the interest of clarity. For each polymorph the zero of energy is taken as the respective Fermi energy.

We plot this in Fig. 5, along with band 20 and 21 from the tetragonal phase. The DOS for the hexagonal band 20 is narrow and sharply peaked, while that of band 21 is much broader and shorter. Indeed, the bandwidth of band 20 is just 0.054 Ryd while that of band 21 is 0.123 Ryd, or more than twice as large. This is again related to the shorter Fe-S nearest neighbor distances (relative to Fe-Fe nearest neighbors), widening the band width, along with the six-fold Fe-S coordination, relative to the two-fold Fe-Fe coordination. For the tetragonal phase there is no such subdivision of bands of different dimensionality, due to the crystal structure in which c-axis conduction is severely inhibited due to the gap between layers.

We note that the specific *shape* of the hexagonal band 21 DOS in fact bears a strong resemblance to a three-dimensional tight-binding DOS, which has a central plateau, while the tetragonal band 20 and band 21 DOS both bear a resemblance to a 2D tight-binding DOS, which is strongly peaked in the center of the band. It is of interest that the tetragonal band DOS curves have long “tails” and in fact much larger bandwidths than the hexagonal DOS. This is surprising given that in a tight-binding scenario larger dimensionalities have *larger* bandwidths (for given hopping integral *t*), but the larger Fe-Fe distances in the hexagonal structure, and the sensitivity of *t* to the exact nearest-neighbor distances, ultimately narrow the bands in the hexagonal phase of FeS.

**Figure 6:**
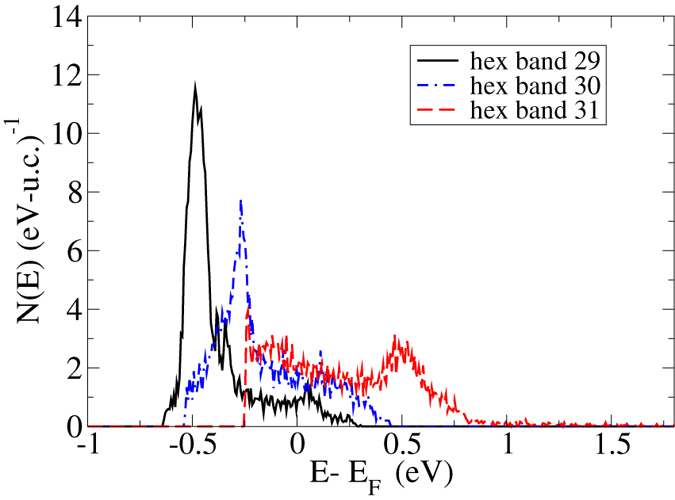
The calculated densities-of-states for the individual bands of non-magnetic hexagonal FeTe, as indicated. The tetragonal results are not shown.

It is of interest to compare these FeS results to those of FeTe, which we present in Fig. 6. For clarity we omit the well-known tetragonal band structure and focus on the hexagonal structure. We use the same vertical (DOS) and horizontal (energy) scale as in Fig. 5 to permit a direct comparison. Unlike in FeS, here we depict *three* hexagonal bands (there is a fourth, not shown, with much lower Fermi-level DOS). These bands are all much broader than in FeS - bands 29 and 30 each have a bandwidth of an eV, whereas band 20 in FeS has only an 0.6 eV bandwidth. (Note that the 10 4*d* electrons per Te are taken as valence but lie in fact far below E_*F*_, so that FeTe band 30 corresponds to FeS band 20). For this reason, the van Hove singularities are much reduced - whereas in FeS this reaches a height of 13.5 per eV, here the heights are 12 and 8 respectively, so that the contribution at E_*F*_ is also much smaller. In addition, the three-dimensional band 31 has a much increased bandwidth, at 1.1 eV (exclusive of the “tails”), than the 0.6 eV bandwidth of FeS band 21. All these factors together mean that hexagonal FeTe is much less magnetic than hexagonal FeS and FeSe, and in fact our calculations find only very marginal magnetic instabilities for hexagonal FeTe, with ordering energies of order an meV per Fe.

Given the superconductivity observed in *tetragonal* FeTe, the question then arises: is *hexagonal* FeTe likely to show superconductivity? We first note the likely presence of non-stoichiometry in hexagonal FeTe, as in the tetragonal polymorph, which could affect the magnetic behavior slightly, although the relevant van Hove singularities, as noted, are not nearly as prominent here. In addition, we note that it is difficult to obtain *unconventional* superconductivity with a substantial T_*c*_ in a hexagonal material. While there are numerous examples of hexagonal superconductors with T_*c*_ above 5 K, the most famous being MgB_2_ (ref. 16) and previously the tungsten bronzes^17^, these are all conventional, electron-phonon mediated superconductors. The hexagonal Fermi surface topology makes magnetically-mediated spin-singlet superconductivity rather unlikely, due to the need for an order parameter sign change under a 60° rotation. A *triplet* state remains possible, although triplet superconductors generally have rather low T_*c*_ values.

To summarize, we have here presented electronic structure calculations of the hexagonal polymorphs of the iron chalcogenides FeS, FeSe and FeTe. We demonstrate that, and explain why, the NiAs-hexagonal polymorphs of the iron chalcogenides FeS and FeSe are much more magnetic than the tetragonal polymorphs, thus precluding superconductivity. We also find that hexagonal FeTe displays only an incipient magnetism. This means that unconventional magnetically-mediated superconductivity is possible in hexagonal FeTe, although a large T_*c*_ is unlikely.

## Methods

All these results are based on first principles density functional theory calculations using the all-electron, linearized augmented planewave (LAPW) code WIEN2K^18^. We use the generalized gradient approximation (GGA) of Perdew, Burke and Ernzerhof throughout^19^. Sufficient numbers of *k*-points - exceeding 1000 in the full Brillouin zone - were used for all calculations. An *RK*_*max*_ of 7.0 was used for the calculations for FeS and FeSe, and 9.0 for FeTe. Here *RK*_*max*_ is the product of the largest plane-wave expansion wave vector and the smallest “muffin-tin” radius (RMT). These RMTs (in units of Bohr = 0.529177 Å) were as follows: tetragonal and hexagonal FeS, 1.85 for S, 2.26 for Fe; tetragonal FeSe, 2.03 for Se and 2.10 for Fe; hexagonal FeSe, 2.39 for Se, 2.49 for Fe; tetragonal FeTe, 2.20 for Te and Fe; hexagonal FeTe, 2.46 for Te and Fe. Spin-orbit coupling was not included. All calculations used experimental lattice parameters. For the tetragonal calculations, the chalcogen height was relaxed; the hexagonal NiAs structure has no free coordinates.
